# Noisy defects in the high-*T*_c_ superconductor Bi_2_Sr_2_CaCu_2_O_8+*x*_

**DOI:** 10.1038/s41467-019-08518-1

**Published:** 2019-02-01

**Authors:** F. Massee, Y. K. Huang, M. S. Golden, M. Aprili

**Affiliations:** 1 Laboratoire de Physique des Solides (CNRS UMR 8502), Bâtiment 510, Université Paris-Sud/Université Paris-Saclay, 91405 Orsay, France; 2 Institute of Physics, University of Amsterdam, 1098 XH Amsterdam, The Netherlands

## Abstract

Dopants and impurities are crucial in shaping the ground state of host materials: semiconducting technology is based on their ability to donate or trap electrons, and they can even be used to transform insulators into high temperature superconductors. Due to limited time resolution, most atomic-scale studies of the latter materials focussed on the effect of dopants on the electronic properties averaged over time. Here, by using atomic-scale current-noise measurements in optimally doped Bi_2_Sr_2_CaCu_2_O_8+*x*_, we visualize sub-nanometre sized objects where the tunnelling current-noise is enhanced by at least an order of magnitude. We show that these objects are previously undetected oxygen dopants whose ionization and local environment leads to unconventional charge dynamics resulting in correlated tunnelling events. The ionization of these dopants opens up new routes to dynamically control doping at the atomic scale, enabling the direct visualization of local charging on e.g. high-*T*_c_ superconductivity.

## Abstract (web summary)

The effects of dopants in high-temperature superconductors on the surrounding electronic structure give insights into their unconventional microscopic behaviour. Here the authors find a new class of defects that they identify as oxygen dopants whose ionization and local environment induce unusual atomic-scale charge dynamics.

## Introduction

Charging effects by defects and impurities have long been recognised as the leading cause for 1/*f* noise in conducting devices^1^. As the miniaturization of devices requires complete understanding and control of defects and trapping sites, much effort has been put into uncovering the properties of individual defects and their charging behaviour in semiconductors by using local probes such as scanning tunnelling microscopy (STM). Charge dynamics of nanometre-sized junctions with e.g. molecules, nano-crystals and semiconductor quantum dots have been extensively studied^2–8^, as well as charging at atomic-scale sites^9–13^. Thus far, the study of charged defects and their dynamics has mostly concentrated on semiconductor materials. However, many correlated electron systems show a rich phase diagram as a function of impurity doping. Doped materials such as the high temperature superconducting cuprates and related ruthenates, whose parent compounds are Mott insulators, are particularly interesting in this regard as the dopant atoms often reside in otherwise insulating layers—a promising environment for charge dynamics to occur. Locally detecting, and possibly manipulating, these sites would open up a new avenue to study the effect of impurities on the physical properties of the system. Thus far, however, to our knowledge no reports have been made of local dynamic charging effects in any of these systems, which seems to indicate that despite the insulating nature of the layers where the dopant atoms reside, the coupling of the dopants to the continuum conduction or valence bands is still too strong for charging to affect tunnelling on the millisecond time-scales that can typically be addressed with an STM.

Real-time detection of charging and de-charging at time-scales on the order of the single electron tunnelling rate (*τ*), which for typical currents (*I*) of a few hundred picoampere is in the nanosecond range (*τ* = e/*I*), is complicated as cable and stray capacitances limit the bandwidth of conventional STMs. A measurement of fluctuations in the current due to the discreteness of the electron charge, or shot-noise, on the other hand, is directly sensitive to changes in the tunnelling dynamics due to local charging effects—even if these are on the time-scale of the tunnelling process. This is because depending on the exact process, local charging can lead to ordering or bunching of electron tunnelling. This results in a reduction (*F* < 1) or enhancement (*F* > 1), respectively, of the current noise, which for random (i.e. Poissonian, *F* = 1) tunnelling is given by *S*_*I*_ = 2e*IF*, where *S*_*I*_ is the shot-noise power spectral density, e the electron charge, *I* the current and *F* the Fano factor^14,15^. In order to measure shot-noise at the atomic scale on correlated electron systems, whose often weakly van der Waals bound layered structures generally require high junction resistances, circuitry operating in the MHz regime has recently been developed^16,17^.

Here, equipped with our shot-noise-enabled scanning tunnelling microscope, we set out to look for signatures of charging at atomic-scale defects on time-scales on the order of the tunnelling process in the near-optimally doped high temperature superconductor Bi_2_Sr_2_CaCu_2_O_8+*x*_ (Bi2212). We immediately visualize sub-nanometre-sized objects where the tunnelling current noise is enhanced by at least an order of magnitude. From the position, current and energy dependence, we argue that these objects are oxygen dopant atoms that were unaccounted for in previous scanning probe studies, whose local environment leads to charge dynamics that strongly affect the tunnelling mechanism.

## Results

### Conventional oxygen dopants

**Fig. 1:**
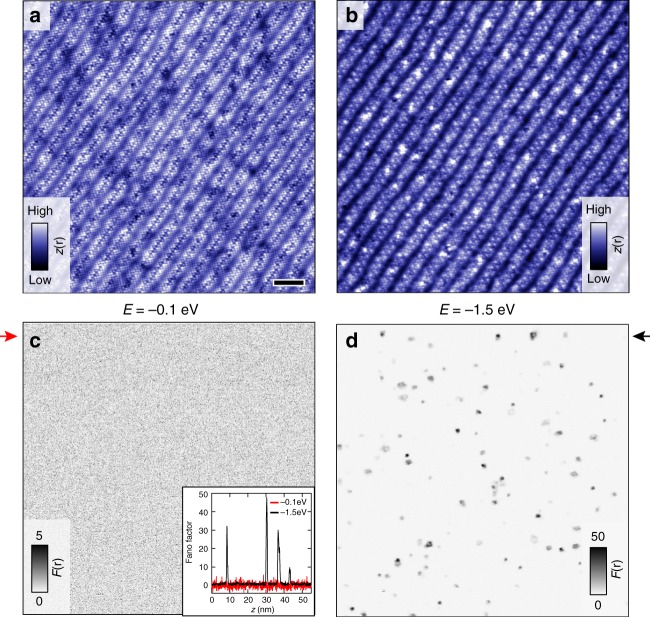
Noisy defects in Bi2212. **a** Constant current image, *z*(**r**), of Bi2212. The scale bar indicates 5 nm, the full field of view is 55 nm (*E* = −100 meV, *I* = 100 pA). **b** Constant current image taken directly after (**a**) with *E* = −1.5 eV, *I* = 400 pA. **c**, **d** Current-noise, or Fano, images (*F*(**r**)) simultaneously taken with (**a**, **b**), respectively. The signal-to-noise for (**c**) is set to be able to resolve changes in the slope of a factor of ~5. This can be greatly improved upon by longer averaging or point spectroscopy, see ref. ^16^. The inset in (**c**) shows a horizontal line cut through (**c**, red trace) and (**d**, black trace) at the position indicated by the arrows. Note: the bright spots in (**b**) do not correspond to the locations with excess current-noise in (**d**), see also Supplementary Note 1

A typical constant current image of cleaved Bi2212 is shown in Fig. 1a, displaying the characteristic incommensurate super-modulation, and clear atomic contrast of the Bi atoms in the BiO surface termination plane. Numerous studies focussing on the tunnelling paths in Bi2212 have attributed this surface appearance to the insight that the main tunnelling path from the (conducting) copper–oxygen plane below the surface through the (insulating) buffer layers to the tip is through the bismuth atoms directly above the copper atoms, whereas the oxygen atoms in the CuO_2_ plane cannot be resolved due to a π-phase difference^18–24^. Upon adding a dopant atom, the situation changes. In general, the interstitial oxygen atoms have a net negative charge from donating one or two holes to the CuO_2_ plane. For sufficiently high positive bias of the tip (negative bias of the sample), this charge can be removed. If the oxygen dopant is coupled strongly enough to the CuO_2_ charge reservoir, the removed electron will be replaced immediately, resulting in the opening of an additional conduction channel. Previous studies have identified two types of such oxygen dopant atoms (at energies *E* = −1 eV and *E* = −1.5 eV), as well as oxygen vacancies (at *E* = +1 eV), through the enhancement of the local density of states^23,25–28^. At the negative sample bias we focus on in this study, we find both types of oxygen dopant and even resolve the previously predicted anisotropic shape for those found at *E* = −1.5 eV^23^, see Supplementary Note 1 and Supplementary Fig. 1. The difference in appearance of the two types of oxygen atoms comes from the observation that although both dopants are below the BiO layer, those found at *E* = −1.5 eV are located in between two Bi atoms, whereas the *E* = −1 eV dopants lie in between a square of four Bi atoms^23,28^. Other than the presence of these dopant states, the general tunnelling process from the CuO_2_ plane to the tip is unaffected: atomic contrast and the super-modulation are nearly identical at *E* = −1.5 eV to that at less negative energy, see Fig. 1b; the additional bright spots correspond to the dopant states that appear at *E* = −1.5 eV.

### Atomic sites with excess current noise

In contrast to the dopant atoms discussed thus far that are strongly coupled to the charge reservoir, local environments are conceivable where this is not the case. Once an electron is removed from such a dopant, it cannot be replaced immediately, leading temporarily to a locally charged (or strictly speaking less charged) state. The charging and de-charging of such a dopant and the accompanying fluctuations in the local potential will completely change the tunnelling dynamics. In general, a strongly fluctuating local potential will have two immediate effects on tunnelling through the dominant current carrying conduction channel(s): the differential conductance will go down as the number of states available for tunnelling is intermittently reduced, and the current-noise will increase as tunnelling will take place in bursts that mimic bunching. Additionally, the current-noise, in analogy with the current dependence of the charging and de-charging of defects that leads to 1/*f* noise, will become quadratically dependent instead of linearly dependent on the current^29^. In fact, in addition to the known (strongly coupled) oxygen dopants, we find defects with spectral features at different energies, a significant number of which display a clear suppression of the density of states. Moreover, unlike the Poissonian shot-noise (*F* = 1) we measure at low energies (see Fig. 1c), at high energy the same locations of atomic dimensions that show additional spectral features display a strongly enhanced current-noise $\left( {F \gg 1} \right)$. The current-noise at these locations, i.e. the dark spots in Fig. 1d, can be up to tens of times higher than for the random tunnelling seen at low energies.

**Fig. 2:**
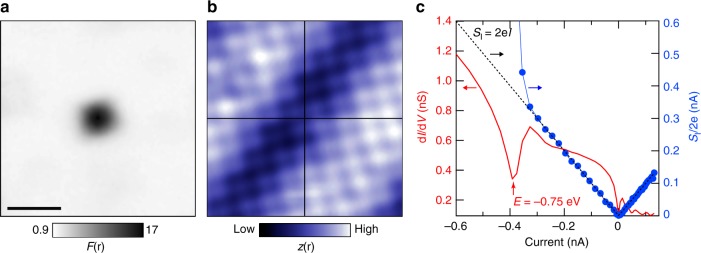
General characteristics of noisy defects. **a** Average current-noise image constructed from 44 defects in Fig. 1d. The scale bar indicates 1 nm. **b** Average topographic image corresponding to (**a**). The clear atomic contrast indicates that the defects are all located at roughly the same location, i.e. in between four Bi atoms. **c** Typical differential conductance spectrum plotted versus the current (solid red, left axis, *E*_setup_ = −1 eV, the dip is located at *E* = −0.75 eV) and simultaneously recorded noise (blue markers and line, right axis): the drop in differential conductance is strongly correlated with the deviation of the noise from Poissonian (dashed line)

Could the defects with excess, or super-Poissonian, current-noise be charging and de-charging dopant atoms? To investigate them in more detail we first focus on their shape and spatial location. In general, given the aforementioned inability to see the surface oxygen atoms, it is difficult to determine whether the noisy defects are in the top BiO layer or below. However, as the images in Fig. 1 show, there is no signature in topography indicating the presence of the noisy defects in or above the topmost BiO layer: the surface Bi atoms are all accounted for, and not unusually distorted due to an otherwise invisible foreign object. Furthermore, their predominant profile in the current-noise is approximately spherical, although their appearance can be clover-like and energy dependent leading to more complicated shapes which we will discuss later. To determine the average shape of the defects and their spatial location, we extract the centre of each defect that is sufficiently isolated from neighbouring ones by fitting an *xy*-symmetric 2D Gaussian. For Fig. 1d, this amounts to 44 defects. We then define an equally sized window around the centre of each defect and construct the average Fano- and topographic images. Figure 2a shows the average defect seen through the current-noise: an isotropic sphere with a diameter of ~8 Å and a magnitude of *F* ~ 17 (at *E* = −1.5 eV). If the defects had been situated at a random *xy*-location in the unit cell, the atomic contrast of the averaged topographic image shown in Fig. 2b would have been washed out. Instead, we maintain clear atomic contrast, indicating that the defects are all located roughly at the same *xy*-location in the unit cell, as expected from the limited number of energetically favourable sites in the unit cell for dopant atoms to occupy^19^. This location, which corresponds to the centre of the images in Fig. 2a, b, is in the middle of a square of Bi atoms—exactly like the oxygen dopant atoms found at −1 eV which have a comparable size and shape^23,27,28^, but no excess current-noise.

To find out more, we turn our attention to spectroscopy. The spatially averaged spectrum of Bi2212 at negative energies is dominated by the appearance of the Cu–O and O bands at *E* ≲ −1 eV^30,31^, with localised peaks in the density of states due to single oxygen dopants^23,25–28^. In agreement with tunnelling affected by local charge modulations, the defects with super-Poissonian noise are generally characterised by a suppression of the differential conductance instead of a peak. This suppression is in most cases preceded by a modest increase at slightly less negative energy, or occurs on the flank of a steep increase. A typical example of a spectrum taken on top of a noisy defect and the simultaneously recorded current-noise is shown in Fig. 2c. Clearly, the deviation from Poissonian noise (i.e. *F* = 1) is strongly correlated with the drop in differential conductance.

### Negative differential conductance

**Fig. 3:**
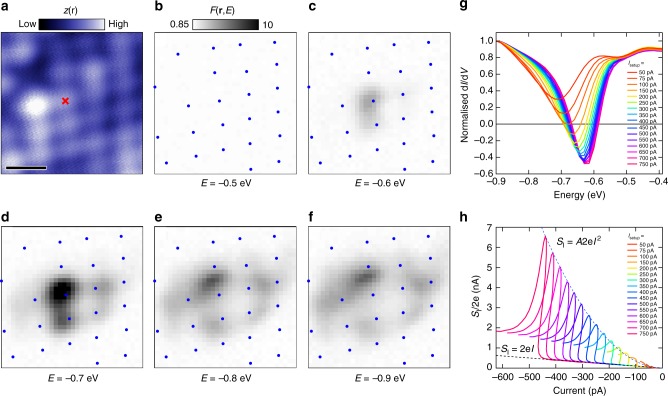
Negative differential conductance and dispersion. **a** Constant current image of an area with a noisy defect (*E* = −100 meV, *I* = 100 pA). The scale bar indicates 0.5 nm. **b**–**f** Spatially resolved current-noise for five different energies: the noise initially appears (in (**c**)) on top of four neighbouring Bi atoms, then radially moves outwards for increasing energies (each energy is recorded at *I*_setup_ = 400 pA). Blue dots mark the location of the Bi atoms in (**a**). **g** Differential conductance taken on the Bi atom marked in (**a**) for increasing setup currents (*E*_setup_ = −1 eV), becoming negative for *I*_setup_ > 100 pA. **h** Current-noise recorded simultaneously with (**g**). Poissonian (*S*_I_ = 2e*I*) and quadratic dependence of the noise on current (*S*_I_ = *A*2e*I*^2^, *A* = 3.4 × 10^10^) are indicated with dotted lines

Although most of the noisy defects we observe on Bi2212 show a modest decrease in differential conductance, a few actually display negative differential conductance (NDC). Instead of a point-like spatial appearance in the centre of a square of four Bi atoms as shown in Fig. 2a, the enhanced noise of these defects is initially located on top of the four neighbouring Bi atoms and radially expands up to one or two lattice sites with increasing energy, see Fig. 3a–f. Point spectra taken on the Bi atom indicated with a cross in Fig. 3a at low currents display similar behaviour to that in Fig. 2c: a slight increase in differential conductance followed by a drop. However, for increasing setup currents, the initial increase is suppressed and for *I*_setup_ > 100 pA the differential conductance becomes negative as can be seen in Fig. 3g. The simultaneously recorded current-noise has a pronounced peak coinciding with the drop in differential conductance. As expected for charge modulated current, the maximum of the current-noise is quadratic in current (Fig. 3h). We further note that even though the current-noise decreases after reaching a peak, it does not return to its Poissonian value, an observation that is true for all noisy defects we have observed.

### Charging and de-charging oxygen dopant

**Fig. 4:**
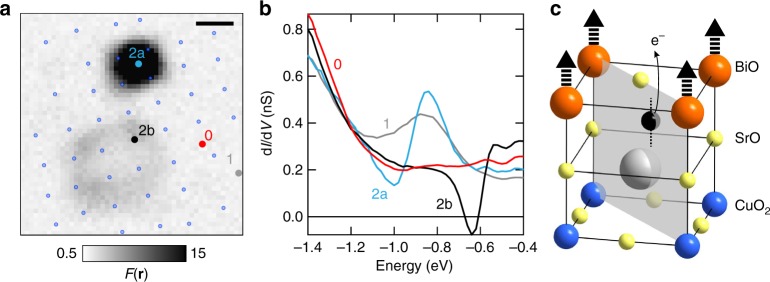
All-in-one: different manifestations of defects on the same *xy*-location. **a** Fano factor on a 3 nm field of view (*E* = −1.4 eV, *I* = 400 pA) with three different defects: one conventional (#1) and two noisy (#2a,b). A location without defect is also marked (#0). Blue dots mark the location of the Bi atoms, the scale bar indicates 0.5 nm. **b** Differential conductance taken at the locations indicated in (**a**). **c** Top three layers of cleaved Bi2212, the dopant location from ref. ^19^ in agreement with our data is shown in black (the dotted line through the dopant indicates possible height variations, not to scale). Weak coupling to the CuO_2_ plane of some defects due to their local environment leads to charging and de-charging that modulates the current through the main conduction channels—for the bottom defect in (**a**), this is through the four neighbouring Bi atoms as indicated by the thick dashed arrows

Given the atomic-scale size a natural explanation for the super-Poissonian noise we observe is that tunnelling is strongly affected by a single charging and de-charging atom. The location of the noisy defects in between four Bi atoms, and their appearance at negative energies in the range −0.5 eV ≤ *E* ≤ −1.0 eV strongly points towards the same oxygen dopants that in most locations lead to a conventional resonance. In this respect, we note that the conventional resonances itself show a spread in energy (−0.8 eV ≤ *E* ≤ −1.0 eV) indicating the presence of different local environments. The noisy defects could therefore very likely be oxygen dopants located in more extreme environments. Figure 4a exemplifies this by showing a Fano image on a 3 nm field of view containing three defects we encounter on the same *xy*-position relative to the unit cell: (1) a conventional one with a resonance around *E* = −1 eV, (2a) a noisy one with the excess current noise appearing directly above the defect, and (2b) a noisy one with excess noise and NDC appearing on the four Bi atoms neighbouring the defect. The spectra corresponding to the three defects, as well as that of a region without one (0), are shown in Fig. 4b. The atomic structure of the topmost three layers of cleaved Bi2212 and the lowest energy position of a dopant atom (black)^19^ in agreement with the location of the defects is shown in Fig. 4c. Although we cannot fully exclude a non-oxygen dopant origin of the noisy defects at this point, all know substitutional defects on the Sr-site, which is the only non-oxygen site in between four Bi atoms between the tip and the CuO_2_ plane, lead to a resonance at positive energies^25^. Additionally, the earlier observation that there are too few sites with a conventional resonance (at *E* = −1 eV and *E* = −1.5 eV) for the expected oxygen doping concentration^27,28^ strongly favours our assignment. We note that since the number of noisy defects is relatively small, *x* ~ 0.005 for Fig. 1d, we do still fall short of the expected number of oxygen dopants for *x* ~ 0.16 even after adding the noisy defects. Due to the limited number, an oxygen doping dependent study of the noisy defects with statistical relevance to further support our assignment will require experimentally challenging large fields of view.

### Importance of local environment

The question is how oxygen dopant atoms, which are located at the same *xy*-location within experimental error, can manifest themselves so dramatically differently. One important clue comes from the current dependence of the defects. In semiconductor systems, a strong dependence of the differential conductance on the setup conditions (i.e. current) is known to be caused by the electric field from the tip that locally bends the bands up- or downwards at the surface, i.e. tip-induced band bending (TIBB)^32^. In essence, for increasing tunnelling currents at the same voltage, the tip is closer to the surface, which increases the tip-induced electric field and reduces the barrier for tunnelling from dopants near the surface to the tip. Crucially, the effect is strongest for dopants closest to the surface. Whereas for metals the screening length is typically too short for such TIBB to play any role, this may not be true in the strongly two-dimensional cuprates, where the *c*-axis transport is semi-conducting down to the lowest temperatures^33^. Furthermore, it is commonly accepted that the screening length in the superconducting state is not substantially different from the normal state value, and may even be longer as has recently been demonstrated in a superconducting thin film transistor^34^. Clearly, the defect with negative resistance (Fig. 3) displays a strong current dependence of the differential conductance and accompanying current-noise. In general, nearly all noisy defects show a finite current dependence, in contrast to the conventional resonances that show no dependence on the setup current (see Supplementary Note 4 and Supplementary Fig. 4). This observation strongly suggests that a key parameter for how a dopant atom reveals itself is its distance from the BiO plane. The distance, combined with the complicated crystal structure, possible nearby elemental substitutions, other defects and vacancies, and the non-commensurate super-modulation, then provides a range of local environments where the coupling of a dopant atom can become weak enough to lead to the array of observed atomic-scale charge dynamics. The fact that the noisy defects and their concomitant features in the density of states typically appear at lower absolute energies than the oxygen dopants that behave conventionally supports this idea. Unfortunately, given the relatively small number of noisy defects, directly correlating their presence to the super-modulation or neighbouring dopants that may provide a sufficiently distorted environment for their charge dynamics to occur is challenging. Even though the super-modulation seems to remain visible in the averaged topography of Fig. 2b, this is not generally the case, see Supplementary Note 2 and Supplementary Fig. 2. Furthermore, noisy defects are apparent in areas with and without conventional dopant, i.e. those that appear as a resonance at *E* = −1.0 eV and *E* = −1.5 eV, see Supplementary Note 1. Additional studies on even larger fields of view, though technically demanding, may provide sufficient statistics for such an analysis.

A consequence of the reduced tunnel barrier for dopants close to the surface due to TIBB is an increase in the rate of ionization. Therefore, for higher setup currents at the same voltage, the average time the dopant is positively (strictly speaking less negatively) charged due to an electron tunnelling to the tip will increase, reducing the differential conductance to lower—or even negative—values and increasing the effective bunching, all in agreement with our observations. Additionally, TIBB is known to produce an energy-dependent halo around charged dopants in semiconductors like GaAs and InAs^10–12^, where the halo reflects the energy-dependent lateral distance from which a dopant can be ionised. The dispersive current-noise in Fig. 3b–f, which has a one-to-one correspondence to changes in the differential conductance (see Supplementary Note 3), is consistent with this mechanism.

Although the general behaviour of the differential conductance—a dip preceded by an increase—and appearance of super-Poissonian noise is very reminiscent of resonant tunnelling in a double barrier structure^35–39^, there is one crucial difference: for resonant tunnelling the noise returns to its Poissonian value once the energy reaches the end of the resonance. This also holds for dynamical Coulomb blockaded tunnelling due to an interacting localised state^40–45^, where a quadratic dependence of the noise on the current has been observed^40^. In contrast, the current-noise of our noisy defects does not return to the Poissonian value of *F* = 1. The fact that we still observe excess current noise at *E* = −1.5 eV in Fig. 1d despite all resonant energies being at lower energies is a direct visualization thereof. Unlike resonant tunnelling, charging and de-charging can still take place at higher energies, resulting in enhanced current-noise even at energies above the resonant energy. The magnitude of the current-noise then reflects the derivative of the differential conductance with respect to the fluctuating potential. As shown in Supplementary Note 5 and Supplementary Fig. 5, we have evidence that this is indeed the case.

## Discussion

Could our observations, instead of the result of charge dynamics at oxygen dopants, somehow be caused by an artefact of the tip, sample contamination, the AC circuitry we use to measure the current-noise, or local heating? To exclude tip effects, we have used tungsten and Pt/Ir tips, both giving identical results. Furthermore, intentionally damaging either tip material has no effect other than reducing the spatial resolution (see Supplementary Note 6 and Supplementary Fig. 7). Multiple as-grown, as well as annealed samples have been studied, all showing similar behaviour, ruling out sample contamination or unusual inhomogeneous samples as culprit. With our ability to completely disconnect our AC circuitry without changing tunnelling conditions, we confirmed that the defect in Fig. 3 displays NDC both with and without AC circuit (see Supplementary Note 6 and Supplementary Fig. 6). Lastly, the noise power spectral density we measure is the sum of the shot-noise and the thermal noise. In principle, local (energy-dependent) heating could therefore enhance the total current-noise. However, to reach noise levels we observe on a number of defects would require local electron temperatures of >20 K, which is highly unlikely for our sample temperature of <2 K.

To summarize, by combining STM and current-noise measurements, we resolve atomic-scale defects where the current-noise is strongly enhanced and the differential conductance reduced—for some defects even negative. These noisy defects most likely constitute a subset of the oxygen dopant atoms that are introduced to turn the Mott insulator Bi2212 into a high temperature superconductor, dopants that were unaccounted for in previous scanning probe studies. The locally enhanced current-noise and reduced differential conductance is expected for charging and de-charging on short time-scales $\left( { \ll \,{\mathrm{ms}}} \right)$ due to weak coupling of these dopants to the CuO_2_ charge reservoir, resulting in local potential fluctuations that strongly affect the dominant tunnelling channel(s). Given the similar *xy*-location, the exact details of the local environment plays a key role in the charge dynamics of oxygen dopants and their effect on the tunnelling process. Their unconventional behaviour, in particular of the defects with NDC, can in principle be utilised to dynamically control the doping at the atomic scale. To this end, finite frequency stroboscopic techniques will be required as the charging and de-charging time-scale is relatively short. Such experiments will enable the direct visualization of the effect of locally changing the charge, providing novel microscopic insight into the origin of the various broken symmetry phases and high-*T*_c_ superconductivity observed in the cuprates.

## Methods

### Samples and measurement details

High quality Bi_2_Sr_2_CaCu_2_O_8+*x*_ single crystals were grown at the University of Amsterdam using the floating zone technique. Both as-grown samples with a *T*_c_ ~ 90 K and samples annealed for 5 days at 450 °C under 20 mbar oxygen pressure with a *T*_c_ ~ 81 K were studied. The samples were mechanically cleaved in cryogenic vacuum at *T* ~ 20 K and directly inserted into the STM head at 4.2 K. Etched atomically sharp and stable tungsten tips, and mechanically cut Pt/Ir tips were used, both with energy independent density of states. Differential conductance measurements throughout used a standard lock-in amplifier with a modulation frequency of 429.7 Hz. STM and simultaneous current-noise measurements were performed with a home-built setup and MHz circuitry, see also ref. ^16^. The Fano factor (*F*) plotted throughout this work is defined as the slope of the current-noise vs. current. For the locations with excess current-noise, where the current-noise is quadratically dependent on the current, the Fano factor at a given current (*I*_0_) is what would have been the slope, had the current-noise at *I*_0_ ≤ *I* ≤ 0 obeyed a linear current dependence. A bandpass filter followed by a Herotek DZM020BB diode was used to integrate the noise amplitude spectral density in the 100 kHz to 5 MHz frequency range. Lock-in measurements of the noise at the LC_cable_ resonance of 1 MHz gave identical results. All presented measurements were recorded at *T* = 1.8 K.

## Supplementary information

Supplementary Information

Peer Review File

## Data Availability

The data that support the findings of this study are available from the corresponding author upon reasonable request.
