# Author Correction: Noisy defects in the high-*T*_c_ superconductor Bi_2_Sr_2_CaCu_2_O_8+*x*_

**DOI:** 10.1038/s41467-019-09696-8

**Published:** 2019-04-03

**Authors:** F. Massee, Y. K. Huang, M. S. Golden, M. Aprili

**Affiliations:** 1 Laboratoire de Physique des Solides (CNRS UMR 8502), Bâtiment 510, Université Paris-Sud/Université Paris-Saclay, 91405 Orsay, France; 2 Institute of Physics, University of Amsterdam, 1098 XH Amsterdam, The Netherlands

Correction to: *Nature Communications* 10.1038/s41467-019-08518-1, published online 1 Feb 2019.

The original version of this Article contained an error in the right-hand *y*-axis of Fig. 2c, which incorrectly read ‘*S*/2*e* (pA)’. The correct version states ‘nA’ in place of ‘pA’. This has been corrected in both the PDF and HTML versions of the Article.

