# Supplementary material for: Noisy defects in the high-Tc superconductor Bi2Sr2CaCu2O8+x
Source: Nat Commun. 2019 Feb 1;10:544. doi: 10.1038/s41467-019-08518-1 (PMC6358600; doi:10.1038/s41467-019-08518-1)
Supplement: Supplementary file 1 — Supplementary Information [file 41467_2019_8518_MOESM1_ESM.pdf]

Supplementary Information to ‘Noisy defects in the high T_c superconductor $Bi_2Sr_2CaCu_2O_{8+x}$’

F. Massee[1,*], Y. K. Huang[2], M. S. Golden[2] & M. Aprili[1]

[1]*Laboratoire de Physique des Solides (CNRS UMR 8502), Bâtiment 510, Université Paris-Sud/Université Paris-Saclay, 91405 Orsay, France*
[2]*Institute of Physics, University of Amsterdam, 1098XH Amsterdam, The Netherlands*

Supplementary Note 1: Dopant location

In line with previous reports[1–3], we find resonant states associated with oxygen dopants at E = -1 V and E = -1.5 eV, as displayed in Supplementary Figure 1. The position of the E = -1 eV states is in between four neighbouring Bi atoms, whereas the E = -1.5 eV states are in between two Bi atoms. Calculations have shown that the strongest intensity for the E = -1.5 eV resonance should be located away from the centre of the defect along the direction of the nearest neighbour Bi atoms[3], leading to two differently oriented resonances depending on the position of the dopant in the unit cell. Experimentally, however, intensity was resolved near only one of the Bi atoms [3]. In our study, we detect intensity on both neighbouring Bi atoms with two possible orientations as can be seen in Supplementary Figure 1b, in perfect agreement with the theoretical prediction. The enlargement of one of the near-horizontally oriented states in the inset of Supplementary Figure 1b clearly shows the directionality of the highest density of states along the nearest neighbour Bi atoms. It is unclear why, unlike Ref. [3], we do experimentally observe the full dumbbell shape, but given the correspondence in energy, the excellent agreement with the theory, and the observation of both possible orientations of the dumbbell, our data fully support the assignment of the oxygen dopant site made by Ref. [3]. In addition to these previously detected resonant states, we find states at a range of energies $<$ -500 meV. Unlike the known resonant states, these additional states show enhanced current-noise. To visualize that these noisy defects are different from the E = -1 eV and E = -1.5 eV states, we plot the Fano image of the main text with the positions of the two types of standard resonances in Supplementary Figure 1c: the noisy defects do not coincide with the known dopants, and can be found in regions both with and without conventional dopants.

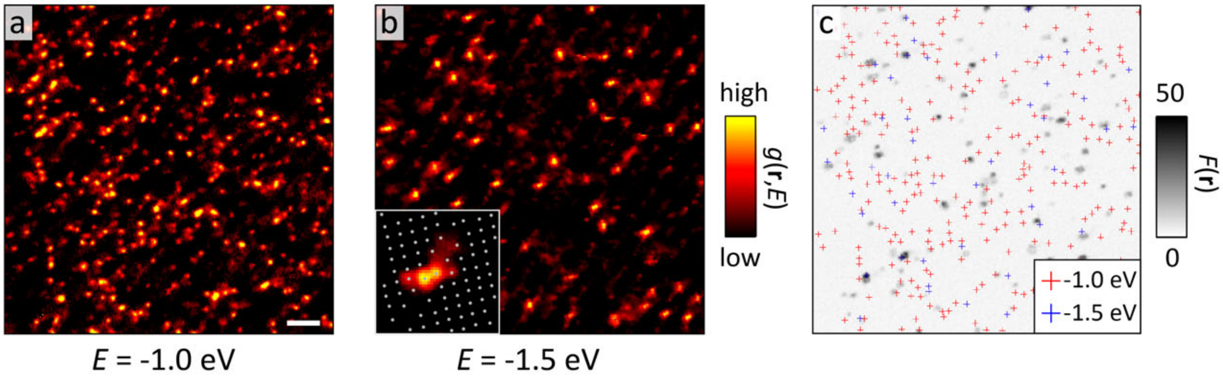

Supplementary Figure 1 Oxygen dopant location. **a,b** Differential conductance, $g(\mathbf{r},E)$), at -1 eV and -1.5 eV, respectively. In the latter, the defects are oriented either near-horizontal or near-vertical. The inset of **b** shows a single near-horizontal defect state with the atomic (Bi) positions indicated with white dots. The scale bar in **a** indicates 5 nm. **c** Fano image (E = -1.5 eV and I = 400 pA) taken at the same location as **a,b**. The noisy defects do not coincide with the known oxygen dopants.

Supplementary Note 2: Average location of the noisy defects

In order to determine the shape and size of the noisy defects, and to find out where they are located in the unit cell, we calculate their spatial average as shown in Fig. 2 of the main text. Supplementary Figure 2 shows an identical analysis for a measurement on a different sample with a different tip material (the full field of view is shown in Supplementary Figure 7f). The size, shape and location of the averaged noisy defects are nearly identical. This strongly supports our assignment of the *xy*-position of the noisy defects, and highlights that we do not observe an artefact, but an intrinsic feature of Bi2212, see also Supplementary Note 6. Secondly, unlike the averaged topography in Fig. 2 of the main text, there are no signatures of the super-modulation in Supplementary Figure 2b, making a clear-cut link between the super-modulation and the noisy defects unlikely. To find out whether there is a correlation between structural distortions caused by e.g. conventional dopant atoms, the super-modulation or a combination, significantly larger fields of view have to be measured for reliable statistics, which is experimentally challenging.

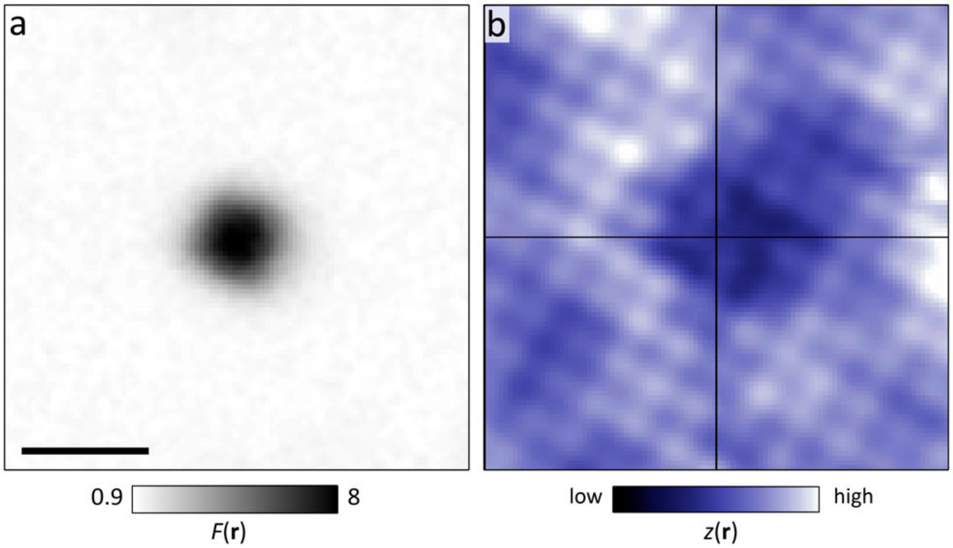

Supplementary Figure 2 Averaged noisy defect for different sample/tip. **a** Average Fano image for the defects in Supplementary Figure 7f, e.g. a different sample and different tip material to that shown in the main text Fig. 2. Nevertheless, the size and shape of the averaged noisy defect are nearly identical (the absolute value is different due to a different setup energy; E = -1 eV, I = 400 pA). The scale bar indicates 1 nm. **b** Averaged topography corresponding to **a**, signatures of the super-modulation are absent.

Supplementary Note 3: Noise and differential conductance

The current-noise and differential conductance are strongly linked to one another as is evident from the point spectrum shown in the main text Fig. 2c. However, the noise gives us a deeper insight into the mechanism underlying changes in the differential conductance that spectroscopy in itself cannot provide. To show how strongly linked the two observables are, not only in energy, but also spatially, we display in Supplementary Figure 3 differential conductance images simultaneously taken with Fano images in the area encompassing that shown in Fig. 3 of the main text. From these images it is immediately evident that the differential conductance and the excess current-noise are strongly linked both in energy and location.

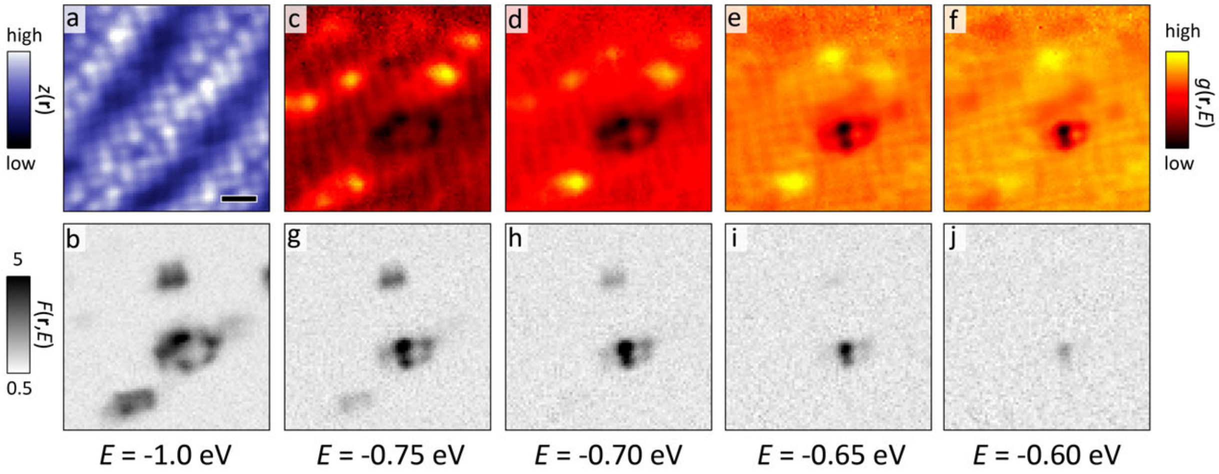

Supplementary Figure 3 Strong link between d*I*/d*V* and current-noise. **a** Constant current image at E = -1 eV and I = 500 pA. The scale bar indicates 1 nm. **b** Fano image simultaneously taken with **a** at the same energy and current as **a**. **c-f** Differential conductance images for a range of energies at the same field of view as **a,b** showing a clearly dispersing signal. **g-j** Fano images simultaneously taken with the d*I*/d*V* image displayed above them, the energy of the Fano image and corresponding differential conductance image is indicated below the Fano images.

Supplementary Note 4: Current dependence of d*I*/d*V*

One important clue as to how oxygen dopant atoms, which are located at the same *xy*-location within experimental error, can lead to very different dynamics is found in the current dependence of the differential conductance. A distinct dependence on the current is a well-known characteristic of tip induced band bending [4], the strength of which depends crucially on the depth of the defect. The defect in Fig. 3 of the main text shows a very strong current dependence, suggesting it is located rather close to the top BiO plane. In general, the differential conductance of defects that show enhanced current-noise right above the defect position have a finite, but less pronounced current dependence - two examples are shown in Supplementary Figure 4a,b. The differential conductance of oxygen states without enhanced current noise do not show an observable current dependence, see Supplementary Figure 4c. The distance of a dopant atom from the BiO plane therefore seems to be an important factor in determining the coupling to the CuO_2 charge reservoir that dictates the presence or absence of charge dynamics.

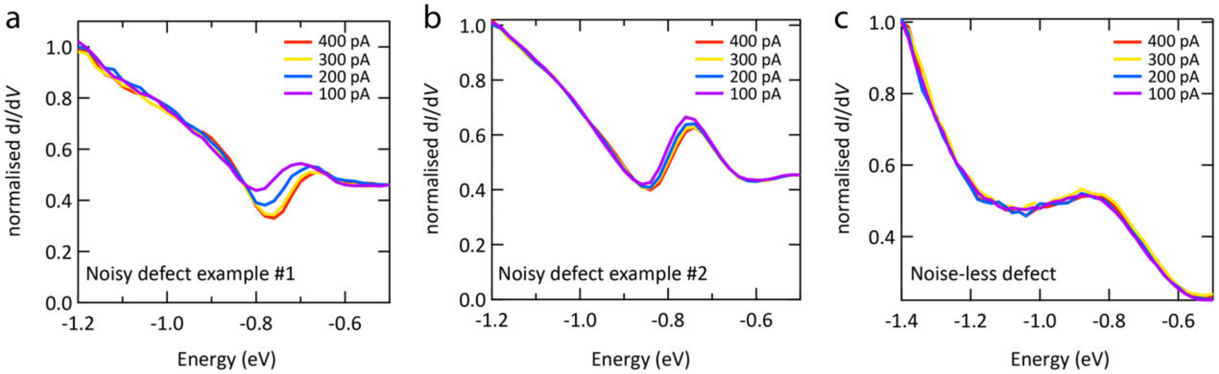

Supplementary Figure 4 Current dependence of d*I*/d*V*. **a** Differential conductance recorded for increasing setup currents for a defects where the enhanced current-noise is located in between four Bi atoms, i.e. right above the defect. **b** Another example of the current dependent d*I*/d*V* of a noisy defect with enhanced current-noise directly above the defect. **c** Measurements on top of a Poissonian defect show no appreciable dependence on the current.

Supplementary Note 5: Voltage dependence of Fano factor at $E > E_{\mathrm{resonance}}$

In the presence of a modulating potential, the excess current-noise will reflect the derivative of the differential conductance with respect to the potential fluctuation. In Supplementary Figure 5a,b we show the differential conductance and its numerical derivative, respectively. Due to the appearance of the CuO_2 bands between E = -1 eV and -1.5 eV, the derivative of the differential conductance strongly increases. At energies above that of the resonance, i.e. $|E| > E_{\mathrm{resonance}}$ instead of returning to the Poissonian value of $F = 1$, the Fano factor is seen to increase, mimicking the d^2I/dV^2 in line with a modulating potential due to charge trapping and de-trapping at the dopant site.

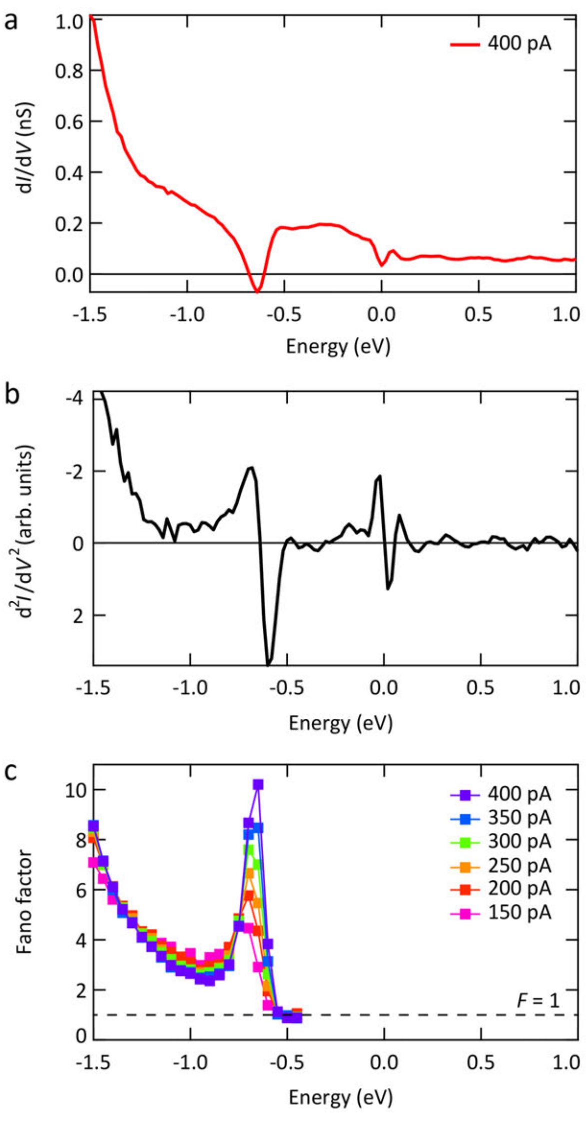

Supplementary Figure 5 Noise vs d^2I/dV^2. **a** Differential conductance at the defect in Fig. 3 of the main text (E_{setup} = -1.5 eV, I_{setup} = 400 pA). **b** Numerical derivative of **a**. **c** Voltage dependence of the Fano factor for a range of currents. At energies above the resonant energy, the voltage dependence strongly mimics the derivative of the differential conductance.

Supplementary Note 6: Circuit and tip independence

To exclude that our observed noisy defects are artefacts due to the AC circuitry, or an unusual tip termination, we performed several tests. Firstly, we can completely disconnect our low temperature AC circuitry with a low temperature switch without altering the measurement conditions. When we do this at a location with negative differential conductance, like that shown in Fig. 3 of the main text, the negative differential conductance and its current dependence persists even without AC circuitry, see Supplementary Figure 6. We can therefore exclude that our circuitry affects our measured signal.

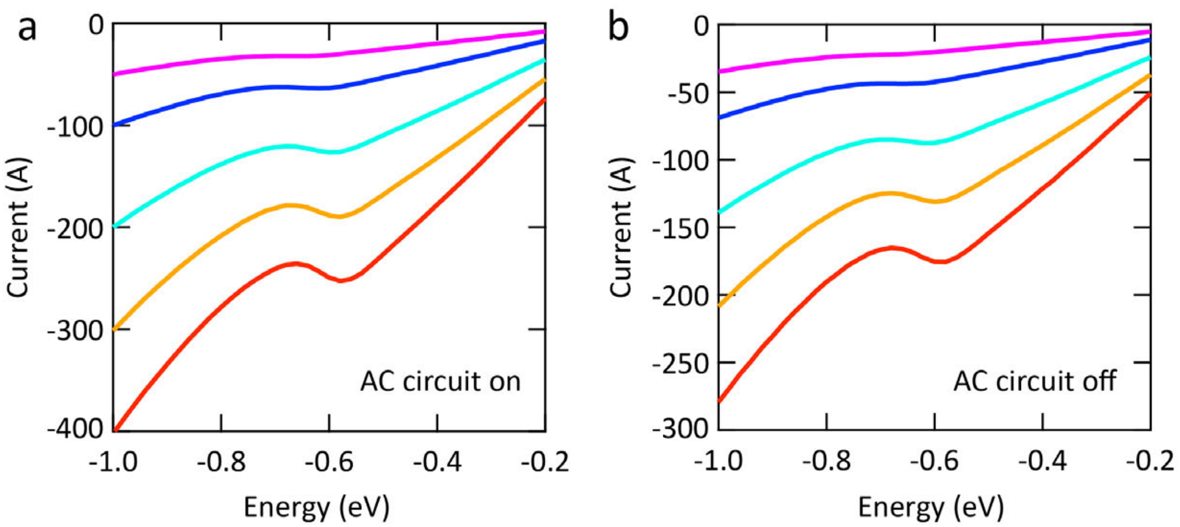

Supplementary Figure 6 AC circuit on/off. **a** Negative resistance measured with AC circuitry for different setup currents on the defect shown in Fig. 3 of the main text (E_{setup} = -1 eV). **b** Spectra on the same location without AC circuitry (E_{setup} = -1.2 eV), proving that the circuitry has no influence on the negative resistance (note that the current scale is different between **a** and **b** due to different set voltages).

To determine whether the excess current-noise we observe is intrinsic to Bi2212 or somehow a tip effect, we intentionally blunted our tip. Supplementary Figure 7a,b show the same field of view before and after blunting, respectively. There is still atomic contrast after tip indentation, but it is heavily blurred and double. The excess current-noise, although reduced in amplitude, is still present (see Supplementary Figure 7c,d). Additionally, we find the same range of atomically sized defects with enhanced current-noise on multiple samples, using different tips, made of a different tip material (W instead of Pt/Ir, see Supplementary Figure 7e,f). We therefore conclude that our observations are not a measurement artefact, but intrinsic properties of tunnelling into Bi2212.

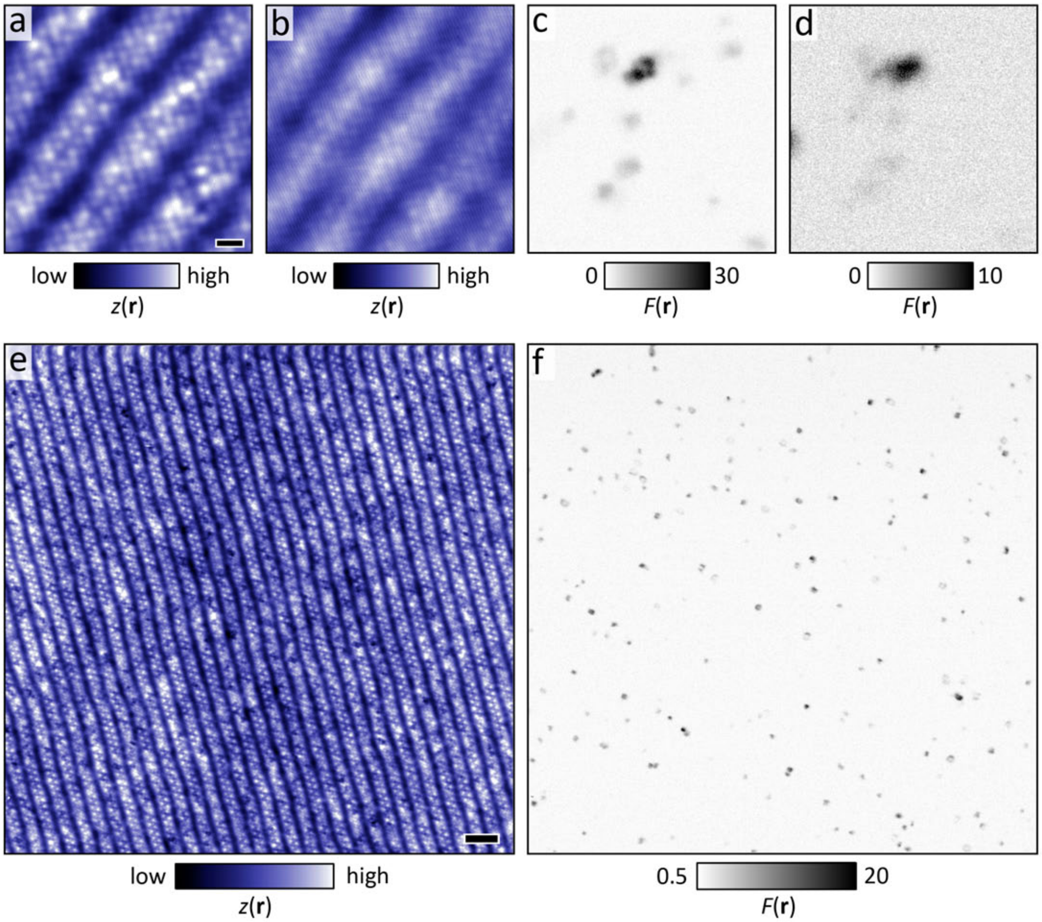

Supplementary Figure 7 Tip apex and material. **a,b** Constant current images on the same field of view with a sharp and blunt tip respectively (E = -1.5 eV, I = 400 pA). The scale bar in **a** indicates 1 nm. **c,d** Fano images corresponding to **a,b**. Although the contrast is blurred with the blunt tip, the excess current-noise is still observed. **e,f** Constant current and Fano image (E = -1 eV, I = 400 pA), respectively, using a W tip on a different sample cleave, showing the same behaviour as with the Pt/Ir tip presented throughout the main text. The scale bar in **e** indicates 5 nm.

References

1. K. McElroy et al., Science **309**, 1048 (2005)
2. I. Zeljkovic et al., Science **337**, 320 (2012)
3. I. Zeljkovic et al., Nano Letters **14**, 6749 (2014)
4. R. M. Feenstra and J. A. Stroscio, J. Vac. Sci. Technol. B **5**, 923 (1987)
